# (2*E*)-1-(2,4-Dichloro­phen­yl)-3-(3,4,5-trimeth­oxy­phen­yl)prop-2-en-1-one

**DOI:** 10.1107/S1600536812016339

**Published:** 2012-04-21

**Authors:** Hoong-Kun Fun, Tze Shyang Chia, M. Sapnakumari, B. Narayana, B. K. Sarojini

**Affiliations:** a X-ray Crystallography Unit, School of Physics, Universiti Sains Malaysia, 11800 USM, Penang, Malaysia; b Department of Studies in Chemistry, Mangalore University, Mangalagangotri 574 199, India; c Department of Chemistry, P. A. College of Engineering, Nadupadavu, Mangalore 574 153, India

## Abstract

In the title compound, C_18_H_16_Cl_2_O_4_, the dihedral angle between the benzene rings is 82.40 (4)°. The meth­oxy groups at both *meta* positions of the 3,4,5-trimeth­oxy­phenyl ring are slightly twisted from the aromatic ring [C—O—C—C = −166.60 (8) and −6.18 (13)°], whereas the meth­oxy group at the *para* position is almost perpendicular [C—O—C—C = 112.08 (9)°]. The ketone O atom is connected to the 2,4-dichloro­phenyl group through a C_ar_—C_ar_—C—O (ar = aromatic) torsion angle of −116.43 (9)°. In the crystal, mol­ecules are linked by C—H⋯O hydrogen bonds into infinite chains along the *b* axis. The crystal structure also features C—H⋯π inter­actions.

## Related literature

For a related structure, see: Fun *et al.* (2012 ▶). For background to various chalcone derivatives, see: Samshuddin *et al.* (2011 ▶). For the stability of the temperature controller used in the data collection, see: Cosier & Glazer (1986 ▶).
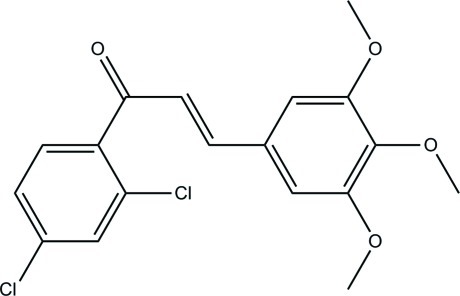


## Experimental

### 

#### Crystal data

C_18_H_16_Cl_2_O_4_
*M* *_r_* = 367.21
Orthorhombic, 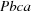

*a* = 9.4305 (5) Å
*b* = 13.9334 (8) Å
*c* = 25.6417 (14) Å
*V* = 3369.3 (3) Å^3^
*Z* = 8
Mo *K*α radiation
μ = 0.40 mm^−1^
*T* = 100 K
0.48 × 0.39 × 0.22 mm

#### Data collection

Bruker APEX DUO CCD diffractometer
Absorption correction: multi-scan (*SADABS*; Bruker, 2009 ▶) *T*_min_ = 0.829, *T*_max_ = 0.917
24763 measured reflections
6139 independent reflections
5445 reflections with *I* > 2σ(*I*)
*R*_int_ = 0.020

#### Refinement

*R*[*F* ^2^ > 2σ(*F* ^2^)] = 0.030
*wR*(*F* ^2^) = 0.086
*S* = 1.03
6139 reflections
220 parameters
H-atom parameters constrained
Δρ_max_ = 0.52 e Å^−3^
Δρ_min_ = −0.22 e Å^−3^

### 

Data collection: *APEX2* (Bruker, 2009 ▶); cell refinement: *SAINT* (Bruker, 2009 ▶); data reduction: *SAINT*; program(s) used to solve structure: *SHELXTL* (Sheldrick, 2008 ▶); program(s) used to refine structure: *SHELXTL*; molecular graphics: *SHELXTL*; software used to prepare material for publication: *SHELXTL* and *PLATON* (Spek, 2009 ▶).

## Supplementary Material

Crystal structure: contains datablock(s) global, I. DOI: 10.1107/S1600536812016339/hb6742sup1.cif

Structure factors: contains datablock(s) I. DOI: 10.1107/S1600536812016339/hb6742Isup2.hkl

Supplementary material file. DOI: 10.1107/S1600536812016339/hb6742Isup3.cml

Additional supplementary materials:  crystallographic information; 3D view; checkCIF report

## Figures and Tables

**Table 1:** Hydrogen-bond geometry (Å, °) *Cg*1 is the centroid of the C10—C15 ring.

*D*—H⋯*A*	*D*—H	H⋯*A*	*D*⋯*A*	*D*—H⋯*A*
C9—H9*A*⋯O3^i^	0.93	2.53	3.3442 (11)	147
C17—H17*A*⋯*Cg*1^ii^	0.96	2.60	3.2965 (11)	130

Symmetry codes: (i) 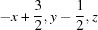; (ii) 
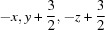
.

**Fig. 1.**
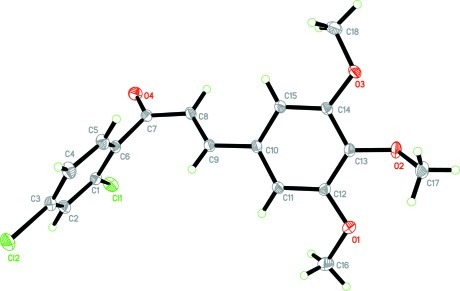
The molecular structure of the title compound with 50% probability displacement ellipsoids.

**Fig. 2.**
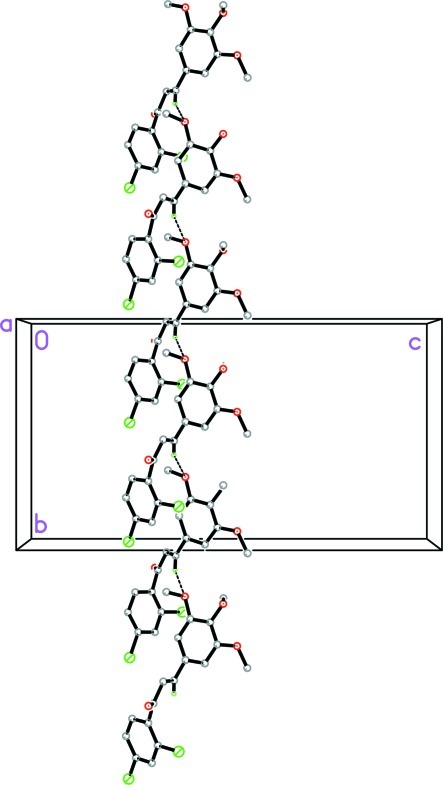
The crystal packing of the title compound. The dashed lines represent the hydrogen bonds.

## supplementary crystallographic information

### Comment

In continuation of our work on the synthesis of chalcones (Fun *et al.*, 2012, Samshuddin *et al.*, 2011) as potential precursors for biodynamic functionalized derivatives, the title compound was prepared and its crystal structure is now reported.

In the title compound (Fig. 1), the dihedral angle between the two benzene rings (C1–C6 & C10–C15) is 82.40 (4)°. The two methoxy groups at both *meta* positions (at atoms C12 & C14) are slightly twisted from the attached benzene ring with torsion angles C16—O1—C12—C13 = -166.60 (8)° and C18—O3—C14—C15 = -6.18 (13)°, whereas the methoxy group at *para* position (at atom C13) is almost perpendicular with C17—O2—C13—C14 = 112.08 (9)°. The atom O4 is connected to 2,4-dichlorophenyl group (Cl1/Cl2/C1–C6) through torsion angle [C5—C6—C7—O4] of -116.43 (9)°. Bond lengths and angles are comparable to a related structure (Fun *et al.*, 2012).

In the crystal (Fig. 2), molecules are linked by C9—H9A—O3 hydrogen bonds into infinite chains along the *b* axis. The crystal is further stabilized by C—H···π interactions (Table 1), involving *Cg*1 which is the centroid of C10—C15 ring.

### Experimental

To a mixture of 2,4-dichloroacetophenone (1.89 g, 0.01 mol) and 3,4,5-trimethoxybenzaldehyde (1.96 g, 0.01 mol) in ethanol (50 ml), 15 ml of 10% sodium hydroxide solution was added and stirred at 0–5 °C for 1 h. The precipitate formed was collected by filtration and purified by recrystallization from ethanol. Colourless blocks were grown from toluene as solvent by slow evaporation method (M.P.: 335–337 K).

### Refinement

All H atoms were positioned geometrically [C—H = 0.93 and 0.96 Å] and refined using a riding model with *U*_iso_(H) = 1.2 or 1.5*U*_eq_(C). A rotating group model was applied to the methyl groups. An outlier (0 0 18) was omitted.

### Crystal data

**Table:**

C_18_H_16_Cl_2_O_4_	*F*(000) = 1520
*M**_r_* = 367.21	*D*_x_ = 1.448 Mg m^−^^3^
Orthorhombic, *P**b**c**a*	Mo *K*α radiation, λ = 0.71073 Å
Hall symbol: -P 2ac 2ab	Cell parameters from 9912 reflections
*a* = 9.4305 (5) Å	θ = 2.7–32.7°
*b* = 13.9334 (8) Å	µ = 0.40 mm^−^^1^
*c* = 25.6417 (14) Å	*T* = 100 K
*V* = 3369.3 (3) Å^3^	Block, colourless
*Z* = 8	0.48 × 0.39 × 0.22 mm

### Data collection

**Table:**

Bruker APEX DUO CCD diffractometer	6139 independent reflections
Radiation source: fine-focus sealed tube	5445 reflections with *I* > 2σ(*I*)
Graphite monochromator	*R*_int_ = 0.020
φ and ω scans	θ_max_ = 32.7°, θ_min_ = 2.7°
Absorption correction: multi-scan (*SADABS*; Bruker, 2009)	*h* = −12→14
*T*_min_ = 0.829, *T*_max_ = 0.917	*k* = −20→21
24763 measured reflections	*l* = −38→35

### Refinement

**Table:**

Refinement on *F*^2^	Primary atom site location: structure-invariant direct methods
Least-squares matrix: full	Secondary atom site location: difference Fourier map
*R*[*F*^2^ > 2σ(*F*^2^)] = 0.030	Hydrogen site location: inferred from neighbouring sites
*wR*(*F*^2^) = 0.086	H-atom parameters constrained
*S* = 1.03	*w* = 1/[σ^2^(*F*_o_^2^) + (0.0458*P*)^2^ + 1.108*P*] where *P* = (*F*_o_^2^ + 2*F*_c_^2^)/3
6139 reflections	(Δ/σ)_max_ = 0.002
220 parameters	Δρ_max_ = 0.52 e Å^−^^3^
0 restraints	Δρ_min_ = −0.22 e Å^−^^3^

### Special details

**Table:**

Experimental. The crystal was placed in the cold stream of an Oxford Cryosystems Cobra open-flow nitrogen cryostat (Cosier & Glazer, 1986) operating at 100.0 (1) K.
Geometry. All e.s.d.'s (except the e.s.d. in the dihedral angle between two l.s. planes) are estimated using the full covariance matrix. The cell e.s.d.'s are taken into account individually in the estimation of e.s.d.'s in distances, angles and torsion angles; correlations between e.s.d.'s in cell parameters are only used when they are defined by crystal symmetry. An approximate (isotropic) treatment of cell e.s.d.'s is used for estimating e.s.d.'s involving l.s. planes.
Refinement. Refinement of *F*^2^ against ALL reflections. The weighted *R*-factor *wR* and goodness of fit *S* are based on *F*^2^, conventional *R*-factors *R* are based on *F*, with *F* set to zero for negative *F*^2^. The threshold expression of *F*^2^ > σ(*F*^2^) is used only for calculating *R*-factors(gt) *etc*. and is not relevant to the choice of reflections for refinement. *R*-factors based on *F*^2^ are statistically about twice as large as those based on *F*, and *R*- factors based on ALL data will be even larger.

### Fractional atomic coordinates and isotropic or equivalent isotropic displacement parameters (Å^2^)

**Table:**

	*x*	*y*	*z*	*U*_iso_*/*U*_eq_	
Cl1	0.28135 (3)	0.217251 (16)	0.611803 (9)	0.02108 (6)	
Cl2	0.68551 (3)	0.053651 (17)	0.730224 (9)	0.02548 (6)	
O1	0.85362 (8)	0.59918 (5)	0.48151 (3)	0.02017 (13)	
O2	0.81150 (7)	0.78269 (5)	0.51372 (3)	0.01807 (13)	
O3	0.67985 (7)	0.82227 (5)	0.60144 (3)	0.01813 (13)	
O4	0.22886 (7)	0.40914 (5)	0.67797 (3)	0.01916 (13)	
C1	0.41001 (9)	0.22749 (6)	0.65990 (3)	0.01458 (14)	
C2	0.48428 (10)	0.14561 (6)	0.67434 (3)	0.01770 (15)	
H2A	0.4638	0.0865	0.6593	0.021*	
C3	0.58992 (10)	0.15424 (6)	0.71183 (3)	0.01775 (16)	
C4	0.62109 (11)	0.24156 (7)	0.73518 (4)	0.02006 (17)	
H4A	0.6907	0.2458	0.7608	0.024*	
C5	0.54655 (10)	0.32244 (6)	0.71967 (3)	0.01803 (16)	
H5A	0.5680	0.3815	0.7346	0.022*	
C6	0.43967 (9)	0.31692 (6)	0.68200 (3)	0.01377 (14)	
C7	0.35648 (9)	0.40595 (6)	0.66920 (3)	0.01436 (14)	
C8	0.43334 (9)	0.48838 (6)	0.64771 (3)	0.01531 (14)	
H8A	0.3917	0.5488	0.6498	0.018*	
C9	0.56145 (9)	0.48055 (6)	0.62511 (3)	0.01455 (14)	
H9A	0.6055	0.4208	0.6259	0.017*	
C10	0.63649 (9)	0.55929 (6)	0.59932 (3)	0.01371 (14)	
C11	0.72028 (9)	0.53747 (6)	0.55583 (3)	0.01506 (14)	
H11A	0.7355	0.4739	0.5462	0.018*	
C12	0.78076 (9)	0.61202 (6)	0.52700 (3)	0.01442 (14)	
C13	0.76521 (9)	0.70727 (6)	0.54348 (3)	0.01393 (14)	
C14	0.68727 (9)	0.72740 (6)	0.58881 (3)	0.01376 (14)	
C15	0.62042 (9)	0.65414 (6)	0.61624 (3)	0.01450 (14)	
H15A	0.5657	0.6680	0.6455	0.017*	
C16	0.84445 (12)	0.50620 (8)	0.45782 (4)	0.02523 (19)	
H16A	0.8912	0.5074	0.4246	0.038*	
H16B	0.7466	0.4893	0.4531	0.038*	
H16C	0.8894	0.4597	0.4799	0.038*	
C17	0.96130 (10)	0.79619 (7)	0.51322 (4)	0.02343 (18)	
H17A	0.9849	0.8475	0.4898	0.035*	
H17B	1.0066	0.7382	0.5018	0.035*	
H17C	0.9933	0.8119	0.5477	0.035*	
C18	0.58610 (11)	0.84829 (7)	0.64272 (4)	0.0248 (2)	
H18A	0.5853	0.9168	0.6465	0.037*	
H18B	0.6178	0.8194	0.6746	0.037*	
H18C	0.4921	0.8262	0.6348	0.037*	

### Atomic displacement parameters (Å^2^)

**Table:**

	*U*^11^	*U*^22^	*U*^33^	*U*^12^	*U*^13^	*U*^23^
Cl1	0.02216 (11)	0.01837 (10)	0.02271 (10)	−0.00144 (8)	−0.00764 (8)	−0.00429 (7)
Cl2	0.03293 (13)	0.02033 (11)	0.02319 (11)	0.00754 (9)	−0.00281 (9)	0.00501 (8)
O1	0.0237 (3)	0.0184 (3)	0.0184 (3)	−0.0001 (3)	0.0076 (2)	−0.0007 (2)
O2	0.0143 (3)	0.0170 (3)	0.0229 (3)	−0.0021 (2)	0.0016 (2)	0.0081 (2)
O3	0.0185 (3)	0.0108 (3)	0.0251 (3)	−0.0023 (2)	0.0047 (2)	−0.0020 (2)
O4	0.0144 (3)	0.0186 (3)	0.0245 (3)	−0.0026 (2)	0.0027 (2)	−0.0016 (2)
C1	0.0158 (3)	0.0142 (3)	0.0137 (3)	−0.0028 (3)	−0.0004 (3)	−0.0005 (3)
C2	0.0225 (4)	0.0133 (3)	0.0173 (3)	−0.0006 (3)	0.0001 (3)	−0.0006 (3)
C3	0.0217 (4)	0.0151 (3)	0.0164 (3)	0.0015 (3)	0.0003 (3)	0.0035 (3)
C4	0.0227 (4)	0.0185 (4)	0.0190 (4)	−0.0014 (3)	−0.0058 (3)	0.0018 (3)
C5	0.0210 (4)	0.0147 (3)	0.0184 (4)	−0.0036 (3)	−0.0036 (3)	0.0000 (3)
C6	0.0148 (3)	0.0122 (3)	0.0143 (3)	−0.0027 (3)	0.0010 (3)	0.0011 (2)
C7	0.0153 (3)	0.0132 (3)	0.0146 (3)	−0.0027 (3)	0.0003 (3)	−0.0011 (3)
C8	0.0148 (3)	0.0118 (3)	0.0193 (4)	−0.0006 (3)	0.0017 (3)	0.0018 (3)
C9	0.0148 (3)	0.0122 (3)	0.0167 (3)	−0.0004 (3)	0.0006 (3)	0.0019 (3)
C10	0.0127 (3)	0.0117 (3)	0.0168 (3)	0.0002 (3)	0.0008 (3)	0.0024 (3)
C11	0.0144 (3)	0.0128 (3)	0.0180 (3)	0.0016 (3)	0.0017 (3)	0.0017 (3)
C12	0.0130 (3)	0.0151 (3)	0.0152 (3)	0.0013 (3)	0.0017 (3)	0.0017 (3)
C13	0.0117 (3)	0.0133 (3)	0.0168 (3)	−0.0003 (3)	0.0006 (3)	0.0035 (3)
C14	0.0120 (3)	0.0111 (3)	0.0182 (3)	−0.0006 (3)	−0.0002 (3)	0.0003 (3)
C15	0.0138 (3)	0.0127 (3)	0.0170 (3)	−0.0009 (3)	0.0023 (3)	0.0006 (3)
C16	0.0312 (5)	0.0230 (4)	0.0216 (4)	−0.0004 (4)	0.0065 (4)	−0.0058 (3)
C17	0.0156 (4)	0.0213 (4)	0.0334 (5)	−0.0034 (3)	0.0058 (3)	0.0033 (4)
C18	0.0246 (5)	0.0164 (4)	0.0334 (5)	−0.0007 (3)	0.0092 (4)	−0.0067 (3)

### Geometric parameters (Å, º)

**Table:**

Cl1—C1	1.7360 (9)	C8—H8A	0.9300
Cl2—C3	1.7319 (9)	C9—C10	1.4635 (11)
O1—C12	1.3655 (10)	C9—H9A	0.9300
O1—C16	1.4334 (12)	C10—C15	1.3992 (11)
O2—C13	1.3702 (10)	C10—C11	1.4000 (12)
O2—C17	1.4252 (11)	C11—C12	1.3967 (12)
O3—C14	1.3628 (10)	C11—H11A	0.9300
O3—C18	1.4260 (12)	C12—C13	1.4005 (12)
O4—C7	1.2252 (11)	C13—C14	1.4034 (12)
C1—C2	1.3890 (12)	C14—C15	1.3909 (11)
C1—C6	1.3971 (11)	C15—H15A	0.9300
C2—C3	1.3896 (13)	C16—H16A	0.9600
C2—H2A	0.9300	C16—H16B	0.9600
C3—C4	1.3874 (13)	C16—H16C	0.9600
C4—C5	1.3864 (13)	C17—H17A	0.9600
C4—H4A	0.9300	C17—H17B	0.9600
C5—C6	1.3983 (12)	C17—H17C	0.9600
C5—H5A	0.9300	C18—H18A	0.9600
C6—C7	1.5040 (12)	C18—H18B	0.9600
C7—C8	1.4658 (12)	C18—H18C	0.9600
C8—C9	1.3444 (12)		
			
C12—O1—C16	116.75 (7)	C12—C11—C10	119.38 (8)
C13—O2—C17	114.96 (7)	C12—C11—H11A	120.3
C14—O3—C18	117.06 (7)	C10—C11—H11A	120.3
C2—C1—C6	121.57 (8)	O1—C12—C11	124.07 (8)
C2—C1—Cl1	118.31 (6)	O1—C12—C13	115.76 (7)
C6—C1—Cl1	120.09 (6)	C11—C12—C13	120.15 (8)
C1—C2—C3	118.35 (8)	O2—C13—C12	121.69 (8)
C1—C2—H2A	120.8	O2—C13—C14	118.34 (7)
C3—C2—H2A	120.8	C12—C13—C14	119.62 (7)
C4—C3—C2	121.75 (8)	O3—C14—C15	124.64 (8)
C4—C3—Cl2	118.81 (7)	O3—C14—C13	114.69 (7)
C2—C3—Cl2	119.44 (7)	C15—C14—C13	120.63 (8)
C5—C4—C3	118.79 (8)	C14—C15—C10	119.16 (8)
C5—C4—H4A	120.6	C14—C15—H15A	120.4
C3—C4—H4A	120.6	C10—C15—H15A	120.4
C4—C5—C6	121.26 (8)	O1—C16—H16A	109.5
C4—C5—H5A	119.4	O1—C16—H16B	109.5
C6—C5—H5A	119.4	H16A—C16—H16B	109.5
C1—C6—C5	118.26 (8)	O1—C16—H16C	109.5
C1—C6—C7	122.87 (7)	H16A—C16—H16C	109.5
C5—C6—C7	118.78 (7)	H16B—C16—H16C	109.5
O4—C7—C8	121.76 (8)	O2—C17—H17A	109.5
O4—C7—C6	120.16 (8)	O2—C17—H17B	109.5
C8—C7—C6	118.06 (7)	H17A—C17—H17B	109.5
C9—C8—C7	122.88 (8)	O2—C17—H17C	109.5
C9—C8—H8A	118.6	H17A—C17—H17C	109.5
C7—C8—H8A	118.6	H17B—C17—H17C	109.5
C8—C9—C10	124.64 (8)	O3—C18—H18A	109.5
C8—C9—H9A	117.7	O3—C18—H18B	109.5
C10—C9—H9A	117.7	H18A—C18—H18B	109.5
C15—C10—C11	120.88 (7)	O3—C18—H18C	109.5
C15—C10—C9	121.04 (7)	H18A—C18—H18C	109.5
C11—C10—C9	118.03 (7)	H18B—C18—H18C	109.5
			
C6—C1—C2—C3	0.19 (13)	C15—C10—C11—C12	−4.30 (13)
Cl1—C1—C2—C3	178.29 (7)	C9—C10—C11—C12	173.10 (8)
C1—C2—C3—C4	0.71 (14)	C16—O1—C12—C11	11.64 (13)
C1—C2—C3—Cl2	−179.40 (7)	C16—O1—C12—C13	−166.60 (8)
C2—C3—C4—C5	−1.43 (14)	C10—C11—C12—O1	−174.04 (8)
Cl2—C3—C4—C5	178.69 (7)	C10—C11—C12—C13	4.13 (13)
C3—C4—C5—C6	1.26 (14)	C17—O2—C13—C12	−74.72 (11)
C2—C1—C6—C5	−0.34 (13)	C17—O2—C13—C14	112.08 (9)
Cl1—C1—C6—C5	−178.41 (7)	O1—C12—C13—O2	4.53 (12)
C2—C1—C6—C7	−176.86 (8)	C11—C12—C13—O2	−173.79 (8)
Cl1—C1—C6—C7	5.07 (11)	O1—C12—C13—C14	177.64 (8)
C4—C5—C6—C1	−0.39 (13)	C11—C12—C13—C14	−0.67 (13)
C4—C5—C6—C7	176.27 (8)	C18—O3—C14—C15	−6.18 (13)
C1—C6—C7—O4	60.07 (12)	C18—O3—C14—C13	171.48 (8)
C5—C6—C7—O4	−116.43 (9)	O2—C13—C14—O3	−7.14 (11)
C1—C6—C7—C8	−121.77 (9)	C12—C13—C14—O3	179.52 (8)
C5—C6—C7—C8	61.73 (11)	O2—C13—C14—C15	170.62 (8)
O4—C7—C8—C9	−162.01 (9)	C12—C13—C14—C15	−2.72 (13)
C6—C7—C8—C9	19.86 (12)	O3—C14—C15—C10	−179.90 (8)
C7—C8—C9—C10	174.62 (8)	C13—C14—C15—C10	2.57 (13)
C8—C9—C10—C15	31.54 (13)	C11—C10—C15—C14	0.95 (13)
C8—C9—C10—C11	−145.86 (9)	C9—C10—C15—C14	−176.36 (8)

### Hydrogen-bond geometry (Å, º)

*Cg*1 is the centroid of the C10—C15 ring.

**Table:**

*D*—H···*A*	*D*—H	H···*A*	*D*···*A*	*D*—H···*A*
C9—H9*A*···O3^i^	0.93	2.53	3.3442 (11)	147
C17—H17*A*···*Cg*1^ii^	0.96	2.60	3.2965 (11)	130

Symmetry codes: (i) −*x*+3/2, *y*−1/2, *z*; (ii) −*x*, *y*+3/2, −*z*+3/2.
